# Support Among Middle School and High School Students for Smoke-free Policies, North Carolina, 2009

**DOI:** 10.5888/pcd10.120135

**Published:** 2013-01-03

**Authors:** Kelly L. Kandra, Anna McCullough, Leah Ranney, Adam O. Goldstein

**Affiliations:** Author Affiliations: Anna McCullough, Leah Ranney, Adam O. Goldstein, University of North Carolina at Chapel Hill, Chapel Hill, North Carolina.

## Abstract

**Introduction:**

In the United States, little is known about youth attitudes toward smoke-free policies. Our research measures North Carolina middle school and high school students’ opinions about smoke-free policies in indoor and outdoor public places as well as private places such as vehicles, homes, and work environments.

**Methods:**

Data come from the 2009 North Carolina Youth Tobacco Survey. The overall middle school response rate was 79.2% (n = 3,805 students); the overall high school response rate was 78.2% (n = 3,301 students). To account for the complex survey design and sampling weights, data were analyzed by using SAS survey procedures.

**Results:**

Most middle school and high school students support smoke-free policies across all venues. Support for smoke-free policies for several venues is also strong among high school students who are current smokers and those who want to stop smoking.

**Conclusion:**

Until smoke-free legislation becomes universal, youth are at risk for exposure in many venues. The North Carolina legislature can protect the health and well-being of North Carolina youth by passing new legislation that is concordant with youth preferences regarding smoke-free policies.

## Introduction

Research suggests that no level of exposure to secondhand smoke (SHS) is safe, and exposure to SHS can damage health immediately and in the long term (1,2). In a 1-year period, an estimated 88 million nonsmokers (aged ≥3 y) in the United States were exposed to SHS; youth aged 12 to 19 years have a higher prevalence of exposure than adults (aged ≥20) (3).

As the evidence of the serious health consequences of SHS exposure continues to grow, more state and local entities are enacting legislation and ordinances to limit exposure. However, most state-level legislation involves smoking restrictions in workplaces, restaurants, and bars (4), venues where smoking restrictions may not necessarily have a strong effect on youth aged 12 to 19. In 2004 in the United States, 41.1% of youth aged 13 to 15 reported SHS exposure in the home, and 54.9% reported SHS exposure in public places (5). From 2000 to 2009, middle school and high school students in the United States have reported significantly less exposure to SHS in vehicles; however, more than 20% of nonsmoking students are still exposed to SHS in a vehicle on a regular basis (6).

Despite high youth SHS exposure in various venues, very little is known about how youth feel about smoking in these venues. Results from the Global Youth Tobacco Survey (GYTS) showed most smoking and nonsmoking youth aged 13 to 15 favored smoke-free policies in public places (7). However, these results are limited in that public places included both indoor and outdoor venues in the same question (7). Furthermore, this finding excludes youth from the United States, as this question was not included in the 2004 US administration of the GYTS (5,8). Our research was designed to assess the opinions of North Carolina middle school and high school students on smoke-free policies for venues in which they are likely to be exposed to SHS (home, vehicles, indoor work environment, indoor public place, and outdoor public place).

## Methods

### Questionnaire

The 2009 North Carolina Youth Tobacco Survey (NCYTS) consisted of 80 questions assessing behaviors, intentions, and attitudes toward various forms of tobacco use. Of the 80 questions on the survey, 68 were core questions from the national Youth Tobacco Survey sponsored by the Centers for Disease Control and Prevention (CDC), and 12 were written specifically for the NCYTS by stakeholder groups for tobacco use control in North Carolina. Our research focused on 5 questions assessing support for limiting exposure to SHS in 5 different venues: homes, vehicles, indoor work environments, indoor public places, and outdoor public places (Table 1). Although the first 4 questions of interest are CDC core questions, the outdoor public places question is specific to the 2009 NCYTS.

**Table 1:** Questions Assessing Support for Limiting Exposure to Secondhand Smoke, 2009 North Carolina Youth Tobacco Survey

Question	Responses
What do you think people should do about smoking inside their home? People should . . .	a. Never allow smoking inside their home
	b. Allow smoking at some times or in some places
	c. Always allow smoking inside their home
What do you think people should do about smoking in their vehicles? People should . . .	a. Never allow smoking in their vehicles
	b. Allow smoking at some times in their vehicles
	c. Always allow smoking in their vehicles
What do you think employers should do about smoking in indoor areas in places where people work? Employers should . . .	a. Never allow smoking in places where people work
	b. Allow smoking only at some times or in some places
	c. Always allow smoking in places where people work
Now think about indoor public places such as malls, movie theaters, clubs or restaurants. Which of these best describes what you think about smoking in indoor public places? Smoking should . . .	a. Never be allowed in indoor public places
	b. Be allowed in indoor public places but only at some times or in some areas
	c. Always be allowed in indoor public places
Now think about exposure to secondhand smoke in outdoor public places such as parks, outdoor sections of restaurants, fairs, or outdoor stadiums. Which of these best describes what you think about secondhand smoke exposure in outdoor public places? Exposure to secondhand smoke should . . .	a. Not be allowed in these types of outdoor public places
	b. Be allowed in these types of outdoor public places but only at some times or in some areas
	c. Be allowed anywhere in these types of outdoor places

We also used 2 other questions from the 2009 NCYTS in this research. “During the past 30 days, on how many days did you smoke cigarettes?” was used to determine current smoking status. Youth who selected answers indicating 1 or more days were classified as current smokers. “Do you want to stop smoking cigarettes?” was used to measure interest in quitting. Response options were “I do not smoke now,” “yes,” and “no.”

### Sampling

The data used in this research come from the fall 2009 administration of the NCYTS, a biannual survey of North Carolina middle school youth (6th–8th grade) and high school youth (9th–12th grade). All student participation was voluntary and anonymous and followed school parental permission procedures. The sampling frame for the drawn sample consisted of all public and charter schools in North Carolina. A multistage cluster sample design in 3 distinct regions of the state (west, central, and east) was used; the “school district” served as the primary sampling unit and “school” served as the secondary sampling unit. Classes were randomly selected within each school, excluding special populations (eg, special needs, English as second language). The 2009 NCYTS middle school district response rate was 97.5%; the response rate of middle school students was 83.5%. The overall middle school response rate was 79.2%; 3,805 middle school students participated and completed the 2009 NCYTS. High school response rates were 97.5%, 83.1%, and 78.2% at the district, student, and overall levels, respectively; 3,301 high school students participated and completed the 2009 NCYTS. Corresponding sampling weights were generated so that results would be representative of all North Carolina middle school and high school youth.

### Analysis

To account for the complex survey design and sampling weights, we analyzed data by using SAS version 9.1 (SAS Institute, Inc, Cary, North Carolina) survey procedures. We used cross-tabulations to generate percentages, 95% confidence intervals (CIs), and χ^2^; we used logistic regression models to generate odds ratios (ORs) and 95% CIs. Specifically, we assessed support for limiting exposure to SHS at 5 different venues by smoking status (current smokers vs nonsmokers) and interest in quitting (smokers who want to stop smoking vs those who do not). We also used smoking status and interest in quitting to predict support for never allowing smoking in the 5 venues. Control variables used for these logistic regression models were sex, race, and currently living with someone who smokes cigarettes. Significance was set at *P* < .05. Youth with complete data across all relevant variables were included in the analyses with the exception of the logistic regression model involving interest in quitting. For this model, youth who responded “I do not smoke now” were excluded. Also, because of the low number of middle school students classified as current smokers (4.3%), only data from high school students were used for the logistic regression models. Table 2 provides an overall demographic description of the middle school and high school students who completed the 2009 NCYTS and a breakdown of the control and predictor variables used in the logistic regression models.

**Table 2:** Demographic Characteristics of Middle School and High School Students Completing the 2009 North Carolina Youth Tobacco Survey

Variable	Middle School Students, % (n = 3,805)	High School Students, % (n *=* 3,301)
**Sex**		
Female	48.7	48.3
Male	51.3	51.7
**Race^a^**		
Minority	44.6	42.4
Nonminority	55.4	57.6
**Live with smoker**		
No	60.0	39.0
Yes	40.0	61.0
**Current smoker^b^**		
No	95.7	83.3
Yes	4.3	16.7
**Want to stop smoking^c^**		
I do not smoke now	94.5	84.2^d^
No	2.8	6.8
Yes	2.7	9.0

a Minority students were defined as students who self-identified as American Indian or Alaska Native, Asian, black or African American, Hispanic or Latino, and Native Hawaiian or other Pacific Islander. Nonminority students were defined as students who self-identified as white.

b Given the low percentage of middle school students classified as current smokers, they were excluded from logistic regression models.

c Given the low percentage of middle school students classified as wanting to stop smoking, they were excluded from logistic regression models.

d High school students who answered “I do not smoke now” were excluded from logistic regression analyses; valid percentages for “no” and “yes” responses are 57.0% and 43.0%, respectively, n = 533.

## Results

Most middle school and high school students surveyed support limiting exposure to SHS at all venues, although responses varied significantly by venue. For middle school students, support for “never allow smoking” was strongest for indoor work environments (85.8%; 95% CI, 83.7–87.9), followed by homes (83.5%; 95% CI, 81.6–85.4), vehicles (82.2%; 95% CI, 79.8–84.6), indoor public places (79.4%; 95% CI, 76.7–82.0), and outdoor public places (63.1%; 95% CI, 61.0–65.3). For high school students, support for “never allow smoking” was strongest for homes (79.4%; 95% CI, 77.2–81.6), followed by indoor work environments (78.9%; 95% CI, 77.3–80.6), indoor public places (75.5%; 95% CI, 73.5–77.4), and vehicles (72.6%; 95% CI, 69.9–75.3), whereas 54.1% (95% CI, 51.6–56.5) of the students believe smoking should never be allowed in outdoor public places.

Nonsmoking high school students supported “never allow smoking” in the 5 venues more strongly than high school students who currently smoke cigarettes (Table 3). Compared with current smokers, nonsmokers were significantly more likely to support “never allow smoking” in homes (OR, 4.5; 95% CI, 3.5–5.8), vehicles (OR, 8.6; 95% CI, 6.5–11.5), indoor work environments (OR, 3.5; 95% CI, 2.9–4.4), indoor public places (OR, 3.6; 95% CI, 3.0–4.3), and outdoor public places (OR, 3.7; 95% CI, 2.8–4.9). Comparisons among current smokers indicated that support for “never allow smoking” in homes, indoor work environments, and indoor public places was significantly higher than support for allowing smoking at some times or in some places for each of those venues; support for “never allow smoking” was also significantly higher than support for “always allow” smoking in vehicles, indoor work environments, and indoor public places (Table 3).

**Table 3:** Support for Smoke-Free Policies Among High School Students, by Smoking Status and Venue,^a^ 2009 North Carolina Youth Tobacco Survey

Variable	Vehicle, % (95% CI)	Home, % (95% CI)	Indoor Work Environments, % (95% CI)	Indoor Public Places, % (95% CI)	Outdoor Public Places, % (95% CI)
**Nonsmoker^b^**					
Never allow smoking	81.0 (78.9–83.2)	85.4 (83.2–87.5)	84.1 (82.6–85.7)	81.0 (79.2–82.8)	59.7 (57.7–61.7)
Allow smoking at some times	15.8 (13.7–17.9)	12.5 (10.5–14.5)	14.2 (12.5–15.9)	18.1 (16.2–20.0)	35.3 (33.6–37.1)
Always allow smoking	3.1 (2.4–3.9)	2.1 (1.5–2.8)	1.7 (1.0–2.4)	0.9 (0.6–1.2)	5.0 (3.8–6.2)
**Current smoker^c^**					
Never allow smoking	30.3 (25.1–35.6)	53.4 (48.2–58.6)	55.6 (50.6–60.6)	50.8 (47.4–54.2)	27.4 (21.5–33.4)
Allow smoking at some times	41.2 (36.8–45.7)	34.5 (30.2–38.8)	33.5 (29.3–37.6)	38.6 (35.1–42.1)	45.0 (40.9–49.1)
Always allow smoking	28.4 (24.5–32.4)	12.1 (8.7–15.6)	11.0 (7.3–14.7)	10.6 (7.5–13.6)	27.6 (22.2–32.9)
χ^2^	826.0	214.6	185.2	579.6	402.7

Abbreviation: CI, confidence interval.

a *P* values for all venues were <.001, *df* = 2.

b n = 2,637 for nonsmokers, weighted percentage = 83.3%.

c n = 576 for current smokers, weighted percentage = 16.7%.

High school students who report that they want to stop smoking cigarettes are significantly more likely to support “never allow smoking” in homes, vehicles, indoor work environments, and indoor public places than their peers who do not want to stop smoking. Of these students, 63.4% (95% CI, 56.2–70.7) indicate that smoking should never be allowed in homes, 36.8% (95% CI, 29.4–44.3) in vehicles, 61.3% (95% CI, 52.1–70.5) in indoor work environments, 59.3% (95% CI, 51.8–66.8) in indoor public places, and 29.7% (95% CI, 23.9–35.5) in outdoor public places. Compared with their peers who do not want to stop smoking, high school students who want to stop smoking were more likely to support “never allow smoking” in homes (OR, 2.6; 95% CI, 1.5–4.5), vehicles (OR, 1.5; 95% CI, 1.0–2.3), indoor work environments (OR, 2.0; 95% CI, 1.2–3.4), and indoor public places (OR, 2.0; 95% CI, 1.1–3.7). Wanting to stop smoking did not significantly increase odds for support of “never allow smoking” in outdoor public places. These youth are, however, significantly more likely to support partial smoking bans in outdoor public places (OR, 2.0; 95% CI, 1.2–3.5) compared with their peers who do not want to stop smoking.

## Discussion

Research suggests that implementing smoke-free policies can positively affect youth. SHS exposure in children aged 0 to 17 decreased by 20% to 50% when homes and workplaces went smoke-free, and significant reductions in SHS exposure for youth aged 12 to 17 were observed when restaurants implemented strong smoking regulations compared with weaker smoking regulations (9,10).

Smoke-free policies have also been linked to reduced cigarette use in youth (11). Our finding that high school students who want to stop smoking cigarettes are more likely to support smoke-free policies in indoor public and private places and partial smoking bans in outdoor public places suggests that these students recognize the possible health benefits of smoke-free policies. Other research has shown that implementing a smoke-free home policy is significantly associated with successful quit attempts in adults in the home (12,13). This is especially important given the influence that family members who smoke have on youth (14). Establishing positive, smoke-free adult role models for youth may be both a benefit and a motivating factor for implementing smoke-free policies, as demonstrated by a study showing that this was a primary reason given for support of a tobacco-free parks policy (15).

Our results show that North Carolina middle school and high school students support smoke-free policies regardless of venue. These findings are similar to those reported from international surveys (7) and provide greater detail about youth support, showing that support for smoke-free policies is high for a variety of specific venues: both indoor and outdoor public places as well as personal places such as homes, vehicles, and work environments.

Although high school nonsmokers showed the highest levels of support for restricting exposure to SHS at all venues, high school current smokers reported higher levels of support for total smoke-free policies in homes, indoor work environments, and indoor public places than for partial or no smoke-free policies. Again, these findings are similar to those from international surveys of youth attitudes (7). Additionally, North Carolina youth attitudes are in line with US adult attitudes toward smoke-free policies, particularly with regard to differences between current smokers and nonsmokers (8,15,16).

Despite the evidence that smoke-free policies benefit youth and adults and that youth and adults clearly support smoke-free policies, much work is still needed regarding smoke-free legislation in the United States. Most smoke-free policies implemented in the United States affect indoor work environments, restaurants, and bars. Approximately 36 states have a workplace (including public and private nonhospitality workplace), restaurant (including attached bar area), or bar (including freestanding bars without separately ventilated rooms) smoke-free policy in place (4).

As of July 2012, only 8 states have enacted smoke-free legislation in outdoor public places: Hawaii, Michigan, and Washington State, which prohibit smoking in outdoor dining and bar patio areas; Iowa, which prohibits smoking in outdoor public transit waiting areas and outdoor dining areas; Maine, which prohibits smoking on beaches in its state parks and in outdoor dining and bar patio areas; New York and Wisconsin, which prohibit smoking in outdoor public transit waiting areas; and Oklahoma, which prohibits smoking in all parks and indoor and outdoor areas of zoos (17–21). Four states have enacted smoke-free vehicle laws specifically for the protection of youth: Arkansas, which prohibits smoking in vehicles where youth aged less than 14 years are present; Louisiana, which prohibits smoking in vehicles where youth aged less than 13 are present; California, which prohibits smoking in vehicles where youth aged less than 18 are present; and Maine, which prohibits smoking in vehicles where youth aged less than 16 years old are present (22).

In North Carolina, the only statewide smoke-free legislation prohibits smoking in restaurants and bars (4). This policy was enacted in 2010 (23), and no other statewide smoke-free policies are being discussed in the state legislature (24). The Clean Air Act does not include environmental toxins from cigarettes and will thus have no effect on SHS exposure in the unprotected venues discussed in this research (25). Protecting youth from such exposure must be a priority, not just in restaurants and bars but also in workplaces, homes, vehicles, and outdoor public venues. The North Carolina legislature can protect the health and well-being of North Carolina youth by passing new legislation that is concordant with what these youth want regarding SHS policies.

There are limitations to our findings. First, the NCYTS is a cross-sectional survey; thus, these results should be interpreted for descriptive purposes only and should not be interpreted for causality or directionality. Second, given that the youth surveyed were middle school and high school students in North Carolina, the results may not be generalizable to students in other areas of the United States. Furthermore, the results also may not be generalizable to youth in this age group who are not in school. However, the results are similar to those for youth aged 13 to 15 worldwide and adults across the United States (7,8,15,16). Also, because these results are based on the self-report of middle school and high school students, they are subject to the honesty of the students. Finally, although the middle school and high school students were surveyed on their opinions of smoke-free policies in various places, no questions were asked to assess how important youth consider these policies. Future research should assess middle school and high school students’ opinions on whether smoke-free policies are considered a matter of priority in their communities.

We found that these middle school and high school students want greater protection from SHS exposure. These findings are important given that many youth have little control over their exposure to SHS, which is dependent on the smokers around them and the degree to which smoke-free policies exist in the places they frequent. Advocates for controlling tobacco use should consider ways to capture and leverage youth’s strong support of smoke-free policies in their legislative advocacy efforts. Policy makers at the state and local levels should enact legislation that protects youth from SHS exposure in many different venues.
